# Patient-Reported Health Outcomes Among Older Adults Undergoing Total Knee Arthroplasty

**DOI:** 10.1093/geroni/igaa057.1219

**Published:** 2020-12-16

**Authors:** Suttiwan Chawengkiattikul, Suparb Aree-Ue, Phichpraorn Youngcharoen, Viroj Kawinwonggowit

**Affiliations:** 1 Faculty of Medicine Ramathibodi Hospital, Mahidol University, BKK, Thailand; 2 Mahidol, Bangkok, Thailand; 3 Mahidol University, BKK, Thailand; 4 Mahidol University, Bangkok, Thailand

## Abstract

Backgrounds Total knee arthroplasty (TKA) is a remedy treatment for severe knee osteoarthritis; yet, postoperative outcomes vary. Preoperative patients’ expectations to functional abilities are important factors influencing postoperative outcomes and satisfaction. Objectives To investigate the association among preoperative patients’ expectations, post-operative functional abilities, and satisfaction to functional abilities among older adults undergoing TKA at 6-week after surgery. Methods Participants were 97 older adults who purposely selected based on the inclusion criteria. The data were collected at preoperative and postoperative TKA by using the Hospital for Special Surgery Knee Replacement Expectations Survey and the Knee and Osteoarthritis Outcome Score - function in daily living. The data analysis was performed by using descriptive statistics, paired t-test, and Pearson product moment correlation coefficient. Results Before surgery, patients’ expectations to postoperative functional abilities had a high level with the total mean score of 70.21(SD =13.86). At 6-week after surgery, the overall functional ability had a significant improvement (t = -9.229, p = .000). Satisfaction to functional ability also had a high level (Mean ± SD = 71.15 ± 14.73). Patients’ expectations to functional abilities had a significantly low positive correlation to postoperative functional ability and satisfaction (r = .273, p < .05; r = .292, p < .01, respectively). A significant moderate positive correlation between functional abilities and satisfaction to functional abilities was observed (r = .603, p < .01). Conclusion Understanding of expectations may be beneficial in gaining knowledge, paving expectations on possible outcomes, and enhancing the quality of care for these populations.

